# Occupational stress among Norwegian physicians: A literature review of long-term prospective studies 2007–2019

**DOI:** 10.1177/14034948241243164

**Published:** 2024-04-10

**Authors:** Bendik Oftung, Reidar Tyssen

**Affiliations:** Department of Behavioural Medicine, Institute of Basic Medical Sciences, Faculty of Medicine, University of Oslo, Norway

**Keywords:** Physicians, work stress, Norway, cohort studies, review

## Abstract

**Aims:**

There are signs of increased stress at work among Norwegian physicians over the last decades, not least among general practitioners (GPs). In this review, we identify trends in both occupational stress and adverse work-related predictors of such stress and burnout in Norwegian physicians.

**Methods:**

We performed an extensive literature search using MEDLINE, Embase and PsycINFO. We included prospective and repeated cross-sectional studies of work stress among Norwegian physicians published in 2007–2019.

**Results:**

Nine studies with observation periods of 1–20 years were included. Occupational stress (global measure) among all doctors decreased gradually from medical school to 20 years later. The prevalence of an effort–reward imbalance increased fourfold among GPs during the period 2010–2019. Five studies reported higher levels of occupational stress among female physicians than among their male colleagues. Work–home conflict levels increased after graduation until 10 years after leaving medical school and plateaued thereafter. Physicians who graduated in a later cohort reported lower levels of work–home conflict and less workplace violence. Work–home conflict, low colleague support, number of work hours and workload/low autonomy were all independent predictors of occupational stress.

**Conclusions:**

**The reduction in occupational stress during the years after leaving medical school may result from increased competency in clinical work and decreased on-call work. The Co-ordination Act implemented in 2012 may explain the increase in occupational stress among GPs. These findings suggest that both reducing work–home conflict and increasing colleague support are important for doctors’ well-being.**

## Background and aims

Norway has a strong history of studying physician health and work life, but lacks an updated review among this occupational group [1,2]. There is evidence of increasing workplace dissatisfaction among Norwegian physicians [3–5]. Understanding the recent trends in occupational stress in representative samples and identifying the important organizational and work-related predictors of such stress are essential. The changes over time and cohort effects in long-term prospective studies can be helpful for identifying the importance of societal and systemic factors to the physician’s work–life balance. Prospective studies are needed to identify possible risk factors and targets for empirically based prevention and intervention.

Surveys and reports in 2018 among general practitioners (GPs) [4] and hospital doctors [5] in Norway showed substantial work pressures in both groups, particularly with respect to work–home conflict, and international cross-sectional studies have linked such stress to burnout [6,7]. A previous study indicated that job satisfaction decreased among Norwegian GPs from 2010 to 2016/2017 [8]. International studies have reported associations between doctors’ stress or well-being and the quality of patient care [9,10], although these findings are discrepant with respect to observed adverse patient outcomes [11]. In the US, there has been a recent focus on burnout in physicians and in primary health care, especially on the pressures associated with the introduction of electronic health records [12]. Recent studies have also focused on the increased occupational stress resulting from an increasing caseload of multi-morbidity patients in primary health care [13]. The increase in stress in Danish GPs has been reported to influence their observed patient care [14].

The last review on work and health among Norwegian doctors covered the period 1997 to 2007 [15]. This review focused mainly on mental health and found that low work control (autonomy), time pressure and demanding patient work are work-related stressors. Somewhat unexpectedly, there were few sex differences in depressive symptoms early in the career, whereas female physicians reported higher levels of depressive symptoms later in their career. This suggests that female physicians experience more stress from work and life over time during their medical career than do their male colleagues.

The aim of this review was to examine whether occupational stress among Norwegian doctors has increased over time and, if so, whether specific factors at work are associated with – or can even predict – this increase. Such factors may be risks for occupational stress and could be amenable to preventative interventions. We investigated whether there are sex differences in the trends relating to occupational stress and work-related stressors. To identify the changes over time and the possible causal or risk factors, we selected only follow-up studies (prospective studies) in which the levels or factors measured at the baseline were compared with a later outcome. Such temporality is one major requirement for concluding the potential causality according to Bradford Hill [16].

In Norway, unique long-term prospective studies on work and health in medical students and physicians have been ongoing over the past 25 years. These include the Reference Panel Study (from The Norwegian Physician Health Survey) of 2000 physicians approached every second year [17] and NORDOC (the Longitudinal Study of Norwegian Medical Students and Doctors) involving 1052 medical students followed up in seven waves of two cohorts [18]. With this background, we examined the trends and course of perceived occupational stress over the last decades in Norwegian physicians. We focused on identifying the work-related stressors that are linked to or predict such stress after controlling for other factors.

## Methods

We applied the search strings described in detail in the Supplemental material Appendix online to the MEDLINE, Embase and PsycINFO search engines, and identified a total of 841 articles. The search ended on 24 February 2023 and included the period 2007–2022. The last published review of this kind (Work and health in Norwegian physicians) was in 2007 [15] and we believe that the past two years (2020–2022) have been not comparable with earlier years in terms of the pressures at work and stress outside of work because of the coronavirus disease 2019 (COVID-19) pandemic. Consequently, only articles with data up to 2019 were included in this review.

**Figure 1.**
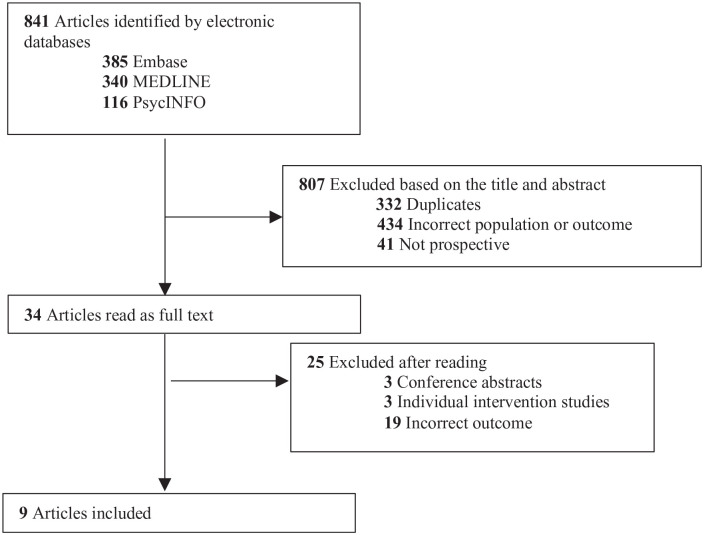
Flow chart of results from the literature search.

We excluded 807 articles, based on the title and abstract (see Figure 1 for more details). We read 34 full-text articles, from which 25 were excluded (Figure 1). Three were conference abstracts, three were intervention studies and 19 were not related to occupational stress (e.g. alcohol problems, anxiety and depression, positive psychology such as satisfaction with life and work). Both reviewers read and selected the full-text articles independently; in the case of disagreement about whether to include an article, the senior author (RT) made the final choice.

The inclusion criteria were prospective and cross-sectional repeated original studies of occupational stress and burnout among physicians working in Norway. The exclusion criteria were reviews, individual intervention studies, studies of medical students, the presence of physical diseases and articles with data gathered after 2019.

The search string in MEDLINE was developed using the PICO tool by a librarian at the University of Oslo Library (Medical Library at The National Hospital ‘Rikshospitalet’) in January 2019; this strategy was then applied to Embase and PsycINFO in January 2020. All three search strings were used for an updated search in February 2023. We created the initial search string to capture studies on job stress, job satisfaction and mental health for a Master’s thesis by the first author, but we found the mental health and positive psychology aspects to be too extensive, and thus focused only on occupational stress for this article.

The term ‘work–home conflict’ has been used in different studies to refer to occupational stress (or outcome) or occupational stressor (or predictor). To avoid confusion, we have clarified this in Table I. The search strings used here are complex constructs that include many subject areas and keywords to capture all relevant articles. Different keywords are used in articles on the same topic, and it is challenging that many articles refer to physicians as professionals and therapists, and not as an occupational group to be studied. Large parts of the electronic search had to be reviewed by hand, and we cannot guarantee that we identified all relevant articles. The search strings are included in the Supplemental Appendix.

**Table I.** Summary of the included studies.

Study, year	Study design (duration); number of time points (TPs)	Sample	*N*	Response rate	Outcome	Trends/course	Work-related stressors or predictors (method)
Røvik et al., 2007 [19]	Prospective (nine years); four TPs	NORDOC 1^a^	631	62%–83%	Work–home conflict (from CJSQ)	Work–home conflict increased in years 1, 4 and 10 after medical school	Lack of reduction in work hours and low colleague support, in addition to female sex, were independent predictors in the fully adjusted model *Adjusted for* personality traits, number of children, spouse support (mixed-model repeated measures)
Langballe et al., 2011 [20]	Prospective (two years); two TPs	Norwegian physicians^b^	1000	52%–68%	Burnout (OLBI)		Work–home conflict and workload predicted burnout. Autonomy and workload were important predictors in men, whereas work–home conflict was most important in women *Adjusted for* having small children, number of hours worked per week, baseline burnout (multiple regression)
Gramstad et al., 2013 [21]	Prospective (four years); three TPs	Junior house officers (University of Bergen)	234	72%–86%	Job stress (CJSQ)		Women experienced more job stress during internship than did men. No work-related stressors identified *Adjusted for* personality traits (multiple regression, structural equation model)
Hertzberg et al., 2016 [22]	Prospective (five years); two TPs	NORDOC 1	293	56%	Burnout (OLBI – emotional exhaustion)	Emotional exhaustion stayed at the same levels in the 10th and 15th years after leaving medical school	Work–home conflict predicted emotional exhaustion in both sexes. The number of working hours and low colleague support were predictive in men *Adjusted for* birth of a child, change in job position, CJSQ factors, support from partner, baseline emotional exhaustion (multiple regression)
Gude and Vaglum, 2017 [23]	Prospective (20 years); five TPs	NORDOC 1	631	55%–75%	Job stress (CJSQ)	Decrease in job stress among all during the 20 years (measured four, 10, 15 and 20 years after leaving medical school)	Female physicians with a physician parent reported higher levels of job stress during the 20 years than did male physicians with a physician parent (ANOVA)
Rosta and Aasland, 2018 [24]	Cross-sectional (1993); prospective (10 years); three TPs	Physician Health Survey^c^, Reference Panel^d^	3608/1499–1612	67%–78%	Perceived bullying in the workplace (at least a few times a month – one item)	Prevalence of bullying was stable from 1993 to 2004 and 2014–2015.	Senior hospital physicians and surgeons experienced more bullying than did other physicians. Female physicians reported more bullying *Adjusted for* job satisfaction, self-rated health, sickness absence (multiple logistic regression)
Hertzberg et al., 2019 [25]	Prospective (10 and 15 years); two TPs	NORDOC 1 and NORDOC 2^e^	1052	57%–67%	Work–home conflict (from CJSQ)	Lower levels of work–home conflict in NORDOC 2 at 10-year follow-up, no difference between the cohorts at 15-year follow-up	Work-related stressors were longer working hours per week and less colleague support. Being a female physician was a predictor in the adjusted model *Adjusted for* number of children, partner support (multiple regression)
Rosta et al., 2020 [26]	Cross-sectional – repeated (2010, 2016 and 2019); three TPs	Reference Panel	1500–2200	67%–72%	Job stress (ERI)	Among GPs (‘*fastleger*’), the prevalence of job stress increased from 10% in 2010 to 40% in 2019. No such increase among physicians employed in hospitals or other positions	
Nøland et al., 2021 [18]	Prospective (20 years); five TPs	NORDOC 1 and NORDOC 2	1052	57%–74%	Violence at work (WPV)	Decreases in both threats and acts over time (four, 10, 15 and 20 years after leaving medical school). Lower rates in NORDOC 2 than in NORDOC 1	Working in psychiatry and being a male physician were predictors of WPV *Adjusted for* personality traits, mental distress, work hours per week, being a GP, job stress (CJSQ) (generalized estimated equations with repeated measures)

Acronyms and measures: CJSQ: Cooper’s Job Stress Questionnaire (modified by Røvik et al. [19]) 43-item (Gude and Vaglum [23]) and 32-item versions (Gramstad et al. [21]), work–home conflict: three items; OLBI: Oldenburg Burnout Inventory – full 16-item Norwegian version (Langballe et al. [20]), OLBI-Emotional exhaustion: eight items (Hertzberg et al. 2016 [22]); ERI: Effort–Reward Imbalance – nine-item short version; WPV: Workplace violence: multiple (twice or more) threats and acts of violence from a patient or visitor.

a NORDOC 1: nationwide cohort of medical students who graduated in Norway in 1993 and 1994 (at all four universities).

b Random sample of 500 male and 500 female physicians drawn from central Norwegian registers of employees (Statistics Norway).

c Physician Health Survey: a random subsample of all Norwegian doctors invited to a postal survey in 1993, nationally representative.

d Reference Panel Study: a randomly selected panel group of 2000 Norwegian doctors approached every second year since 1994 (imbalanced: retired doctors are replaced by young ones), nationally representative.

e NORDOC 2: Nationwide cohort of medical students in Norway who graduated in 1999 (at all four universities).

ANOVA: analysis of variance; GP: general practitioner

## Results

Nine studies met the selection criteria, the findings of which are summarized in Table I [18–26]. The populations included in more studies are described both here and in more detail in the text following the individual studies. The observation periods ranged from one to 20 years. Two studies were about burnout (or emotional exhaustion), two about general job stress, two about work–home conflict, one about effort–reward imbalance, one about bullying/harassment and one about workplace violence.

Five studies used the NORDOC sample, a follow-up survey lasting 20–25 years of two nationwide cohorts of medical students who graduated in 1993/1994 (NORDOC 1, *n* = 631) and 1999 (NORDOC 2, *n* = 421) [27,28]. The students were from all four universities with medical schools in Norway, and the study was directed by the Department of Behavioural Medicine at the University of Oslo.

Two studies used the Reference Panel in The Physician Health Study, a dynamic cohort of physicians that is nearly representative of all Norwegian physicians. The term ‘dynamic cohort’ means that retired physicians have been substituted by young physicians to maintain a representative sample. The panel has been followed up every second year from 1994 (*n* = 1272–2200) [17,29].

Two studies were from other sources (see Table I).

### Trends in occupational stress with time in Norwegian physicians

Three studies reported alterations in perceived work stress with time in the long-term follow-up of representative samples [19,23,26]. The overall perceived job stress decreased from graduation until nine and 20 years later in all physicians [19,23]. A recent study showed a fourfold increase in job stress (effort–reward imbalance) during the period 2010–2019 among Norwegian GPs (‘*fastleger*’), but no such increase was found among physicians in other positions [26]. Five studies found higher levels of occupational stress in female compared with male physicians. This applied to job stress in junior phy-sicians during their internship [21], to perceived bullying in the workplace [24], to the subgroup of physicians with a physician parent [23] and to work–home conflict in the 10th and 15th years after leaving medical school [19,27]. One study found higher rates of workplace violence in male than in female physicians, but this occupational stress decreased over the career [18]. Trends in work–home conflict and emotional exhaustion during long time periods were examined in three studies [19,22,27]. The work–home conflict increased from medical school to the 10th year [19] and then decreased until the 15th year [27]. Physicians who graduated in 1999 (NORDOC 2) reported lower levels of work–home conflict and less workplace violence than did those who graduated in 1993/1994 (NORDOC 1); these associations remained after controlling for other predictors of such stress [18,27]. One study found that emotional exhaustion stayed at the same levels in the NORDOC 1 cohort from the 10th to 15th years after leaving medical school [22].

### Predictors of occupational stress (occupational stressors)

The factor *work–home conflict* predicted emotional exhaustion in two studies [20,22], and this effect was stronger in female doctors in one study [20].

Four studies found that *number of work hours per week*, *workload* and *low autonomy* were predictors of work–home conflict or burnout. [19,20,22,27]. Two of the articles showed that reduction in work hours, workload and autonomy were stronger predictors among male physicians [20,22].

Three studies showed the importance of *colleague support*. Lack of colleague support predicted high levels of work–home conflict in both sexes [19] and emotional exhaustion among men [22]. By contrast, high levels of colleague support predicted lower levels of work–home conflict (from the 10th to 15th years after medical school) [27].

In the analyses that included *specialty and position*, in addition to the increase in effort–reward imbalance in GPs compared with other physicians, working in psychiatry was a predictor of workplace violence in a 20-year follow-up [18]. Senior hospital physicians and surgeons reported more bullying than did other physicians [22].

## Discussion

The major finding of our review is that occupational stress decreased by 20 years after medical school, particularly after the first years of speciality training. The levels and rates of occupational stress seem to be higher among female than among male physicians. One exception is workplace violence, which is reported more often by men. There was a clear increase in occupational stress among GPs in the period from 2010 to 2019. The proportion of GPs (‘*fastleger*’) with effort–reward imbalance increased fourfold during this period. Work–home conflict levels increased during the first years after medical school and then decreased from the 10th to 15th years. Work–home conflict was also an independent predictor because this variable predicts burnout, but one study showed lower levels of this stress in a younger cohort of physicians 10 years after graduation. A high number of work hours per week and perceived workload, and lower levels of autonomy at work and support from colleagues, were identified as factors that predict occupational stress. Some of these factors showed sex differences in their impact.

### What can explain the changes during time periods?

The decrease in work stress during the first years after medical school can be explained by an increase in clinical experience and competency, as well as less demanding on-call work schedules and rosters over time. Other international and Norwegian studies have shown that on-call workload and sleep deprivation are important stress factors during the first postgraduate years, and that such stresses decrease later in the career [30–32].

The large increase in work stress among GPs during the period 2010–2019, without such alterations in other specialities, reflects particular changes in the working conditions in primary health care during this period. This may be linked to the implementation of two major health-care reforms during the past decade, ‘The Co-ordination Reform’ in 2012 and ‘The Future Primary Care – Proximity and Comprehensiveness’ in 2015. Both reforms have increased the transfer of tasks and patients from the hospitals and outpatient clinics to primary care [26] and have thus created more work for the GPs; this has also been shown in recent qualitative studies [33,34]. A governmental evaluation in 2023 of ‘The Regular General Practitioners’ Scheme’ (‘*Fastelegereformen*’) from 2001 showed that GPs during the last decade have experienced a considerable and unmanageable increase in workload [35]. The proportion of GPs with 50 days or more per year with out-of-hours work increased from 10% to above 40% during the years 2010–2021.This increase includes new tasks, an increase in the volume of previous tasks, patients being more demanding than before (e.g. more multi-morbidity patients) and GPs lacking the opportunity to delegate work to others. The increased pressure on GPs can result in a reduced quality of patient care [36]. The re-cent Organization for Economic Cooperation and Development report ‘Health at a glance 2021’ shows that Norway scored relatively low and worse than before on some integrated care quality outcomes, such as re-admittance and mortality within one year after hospital discharge of patients with ischaemic stroke and heart disease. Norway has the second-highest rate of suicide within one year after hospital discharge for mental disorders in Europe (next to the Netherlands).

The initial increase in work–home conflict was expected because this time coincides with the years of establishing a family and childcare. This is an important stress factor among both Norwegian [37] and US physicians [38], but the Norwegian studies are prospective and show such stress as a possible risk factor and cause of burnout [20,22]. It is promising that the later NORDOC cohort experienced lower levels of work–home conflict and less workplace violence. The first may reflect the increased availability of kindergartens in the second compared with the first cohort [39]. The simulation training of incidents with violent patients during recent decades may explain the decreasing trend in workplace violence [18].

The higher level of reported occupational stress among women compared with men is important because of the increasing percentage of women in medicine. This finding among relatively gender-liberal Norwegian physicians has also been reported in international studies, both early [32] and later in the career [40]. This difference supports the notion that the medical career exacts a heavier toll on female physicians. In a previous NORDOC study, the role of personality traits was offered as a possible explanation because trait neuroticism (self-criticism or low self-esteem) was a stronger predictor of job stress during medical internship among women than among men [31]. One study in our review found that work–home conflict was a stronger predictor of burnout in female doctors [20]. However, work–home conflict was initially at the same level in both male and female doctors in the NORDOC studies, and work–home conflict became more prominent among the women only after adjusting for partner support and working hours [19,22]. This means that such stress was buffered by having a supportive partner and reduced work hours for the women. A reduction in work hours and the ability to obtain part-time work may be particularly important for reducing occupational stress in female physicians.

### Important work-related predictors

It was not surprising that the *number of work hours* and *workload* were independently associated with increased job stress. Physicians often work more than 40 hours per week, despite cultural and individual differences [41]. A previous US study found a threshold for reduced well-being at 60 hours per week among oncologists [42], and this is presumably lower among young Norwegian physicians of today [37].

*Low autonomy* is probably a more important detrimental factor for health and well-being than the number of hours at work per se. There have been several studies of Karasek’s demand–control model, in which a high level of demands and low level of autonomy lead to mental health impairment and even cardiovascular disease [43].

A major protective factor against work stress seems to be *colleague support*. A poor working climate and lack of support from colleagues are important stress factors among young physicians [32]. Support from senior colleagues and a good learning climate protect Norwegian and Swedish junior doctors during their internship (as preregistration house officers) [31,44]. Colleague support enhances well-being and life satisfaction among Norwegian physicians later in their careers as well [28].

In our review, *work–home conflict* was both an important outcome as well as a predictor of or possible risk factor for burnout in two of the articles reviewed [20,22]. This is concurrent with international research showing that work–life balance is important for reducing stress and burnout, particularly early in the career [32,40].

### What can improve physician working conditions and reduce their work stress?

Both organizational and individual measures against stress and burnout seem to be necessary [45]. It seems that Norwegian GPs are a group at higher risk of stress, burnout and early retirement. This is not only an issue related to doctors’ responsibility, but a societal and political issue that needs to be solved, for example, by increasing the number of physician positions in primary health to reduce patient lists for each doctor. Physicians may benefit from more support from colleagues, and this is the responsibility of administrators and leadership within health-care services [46]. Support and supervision by experienced colleagues can attenuate work stress among younger doctors [31] and thereby help to ensure the quality of patient care. To avoid unacceptable work–home stress, young physicians should be guaranteed their social security benefits, parental leave, availability of kindergarten and the right to stay home with sick children [37]. The changed gender roles in the later cohort, with greater shares of household chores and childcare between partners, imply a need for more physician positions, and not only among female doctors.

In the NORDOC studies, female physicians re-ported about three hours less a week at work than their male colleagues. This may have implications for the estimates of the number of physician positions in the future because of the increase in the proportion of women in medicine.

Our review has also shown that in addition to a reduction in the number of work hours, important intervention measures include reducing the perceived workload and increasing control (or autonomy) at work, especially among male physicians. Physicians should be offered training in handling threats and acts of violence from patients, and this training should be given early in the medical career.

Counselling for physicians with burnout in the Villa Sana scheme has led to a reduction in emotional exhaustion and work stress in participants at one year, and this change was maintained at a three-year follow-up of the intervention [47,48]. A longer duration of full-time sick leave predicts a reduction in emotional exhaustion when resuming a full workload three years later [49]. For those in high-performance professions, such as medicine, a longer duration of sick leave may be necessary for full restitution [50].

### Strengths and limitations

A major strength of this review is the relatively large number of studies with a prospective and longitudinal design, which is a major requirement for inferring trends with time and possible causality. This is unique internationally. A recent international *JAMA* review of the factors associated with stress and burnout in young physicians found 48 studies, but only four of these were prospective [32]. The measurements used in the prospective studies of our review were similar at every time point of follow-up and are all previously validated instruments. The Reference Panel includes representative data of Norwegian physicians and provides robust data about the changes in trends over time in Norway. Given the comparable political systems and public health services, we believe the results of our findings are representative of other Nordic countries, particularly with regard to the predictors of occupational stressors. Few studies have compared occupational stress levels between countries, but one found higher levels of exhaustion (burnout) among university hospital physicians in Sweden than in Norway and Iceland [51]. We believe that the work-related predictors may be even more important in a private and more profit-driven health-care system, such as in the US.

A limitation of our review is that the included studies used different instruments to measure work stress and burnout, which made comparisons across studies difficult. The effort–reward imbalance was measured using a short nine-item version that has been validated, but the shorter form may have reduced the validity. The original search string was quite broad and included job satisfaction and mental health studies, and we did not use national databases other than those included in MEDLINE. We may therefore have missed some studies. A meta-analysis or systematic review would have provided more comparable estimates of the effects of occupational stressors. Because some of the studies were based on data from the same cohort (e.g. NORDOC 1), the independence and ability to generalize the findings may be limited. The ‘healthy worker effect’ and attrition of participants affected by unhealthy occupational stress may have led to an underestimation of important effects (type 2 error) in our long-term follow-up studies. Another issue is the large immigration of physicians from abroad to Norway during the past two decades, which means that the NORDOC cohorts that have been followed up since the early part of the study (1993) are less representative of Norwegian physicians of today. New cohort studies of the present workforce of physicians are needed in Norway, and this applies in particular to young doctors in training. Finally, there may have been changes in work stress (e.g. among hospital doctors) during the COVID-19 pandemic over the past few years that we have not captured in this review.

## Conclusions

During the period 2010–2019, a large increase in occupational stress (effort–reward imbalance) occu-rred among Norwegian GPs (‘*fastleger*’), and this trend may have resulted from several organizational changes in primary health care. Female physicians seem to be at particular risk of occupational stress. Work–home conflict is an important independent predictor of burnout. Other possible risk factors are an increased number of work hours, increased workload and demands, and decreased professional auto-nomy. Support from colleagues appears to be an important protector against occupational stress.

## Supplemental Material

sj-docx-1-sjp-10.1177_14034948241243164 – Supplemental material for Occupational stress among Norwegian physicians: A literature review of long-term prospective studies 2007–2019 Supplemental material, sj-docx-1-sjp-10.1177_14034948241243164 for Occupational stress among Norwegian physicians: A literature review of long-term prospective studies 2007–2019 by Bendik Oftung and Reidar Tyssen in Scandinavian Journal of Public Health
