# Supplementary material for: Occupational stress among Norwegian physicians: A literature review of long-term prospective studies 2007–2019
Source: Scand J Public Health. 2024 Apr 10;53(3):302–10. doi: 10.1177/14034948241243164 (PMC12012265; doi:10.1177/14034948241243164)
Supplement: sj-docx-1-sjp-10.1177_14034948241243164 – Supplemental material for Occupational stress among Norwegian physicians: A literature review of long-term prospective studies 2007–2019 [file sj-docx-1-sjp-10.1177_14034948241243164.docx]

**Appendix: Search strings**

| Search engine | Search string |
| --- | --- |
| **Embase:** | ((norwegian doctor* or norwegian physician*).mp. or ((norway/ or norway.mp. or norwegian*.mp.) and (physician*.mp. or physician/ or medical doctor*.mp. or medical student/ or medical student*.mp.)) and ((work-life balance/ or social support/ or professional burnout/ or dysthymia/ep or job stress/ or life stress/ or mental stress/ or workload/ or drinking behavior/ep or binge drinking/ep or alcohol intoxication/ep or depression/ep or suicide/ep or mental health/ or malpractice/) or (life satisfaction* or work-related stress* or burnout* or stress* or alcohol drinking* or depression* or anxiety* or suicide* or mental health* or work-life balance* or social support* or mental fatigue* or workload* or binge drinking* or alcoholic intoxication* or physician impairment* or mindfulness* or psychological distress*).tw.)) or (norwegian doctor* or norwegian physician*).mp. or nordoc.mp. |
| **Medline:** | ((norwegian doctor* or norwegian physician*).mp. or ((norway/ or norway.mp. or norwegian*.mp.) and (physician*.mp. or physician/ or medical doctor*.mp. or medical student/ or medical student*.mp.)) and ((Physicians/px or Work-Life Balance/ or Social Support/ or BURNOUT, PROFESSIONAL/ or Mental Fatigue/ or STRESS, PSYCHOLOGICAL/co, ep, px, th or WORKLOAD/px or Alcohol Drinking/ep, px, th or Binge Drinking/ep, px or Alcoholic Intoxication/ep, px or DEPRESSION/ep or SUICIDE/ep, px or Mental Health/ or physician impairment/  ) or (life satisfaction* or work-related stress* or burnout* or stress* or alcohol drinking* or depression* or anxiety* or suicide* or mental health* or work-life balance* or social support* or mental fatigue* or workload* or binge drinking* or alcoholic intoxication* or physician impairment* or mindfulness* or psychological distress*).tw.)) or (norwegian doctor* or norwegian physician*).mp. or nordoc.mp. |
| **PsycINFO** | ((norwegian doctor* or norwegian physician*).mp. or ((norway/ or norway.mp. or norwegian*.mp.) and (physician*.mp. or physician/ or medical doctor*.mp. or medical student/ or medical student*.mp.)) and ((work-life balance/ or social support/ or occupational stress/ or exp Mental Health/ or psychological stress/ or social stress/ or academic stress/ or work load/ or exp Alcohol Drinking Patterns/ or exp Binge Drinking/ or exp Alcohol Abuse/ or exp Alcohol Intoxication/ or "depression (emotion)"/ or major depression/ or exp Suicide/ or impaired professionals/) or (life satisfaction* or work-related stress* or burnout* or stress* or alcohol drinking* or depression* or anxiety* or suicide* or mental health* or work-life balance* or social support* or mental fatigue* or workload* or binge drinking* or alcoholic intoxication* or physician impairment* or mindfulness* or psychological distress*).tw.)) or (norwegian doctor* or norwegian physician*).mp. or nordoc.mp. |
